# Terra Cognita: Using Earth Observing Systems to Understand Our World

**DOI:** 10.1289/ehp.113-a98

**Published:** 2005-02

**Authors:** Charles W. Schmidt

Who would believe that a butterfly, by flapping its wings in Peru, could set off a chain of events leading to a monsoon thousands of miles away? This familiar notion from chaos theory may seem absurd even as it raises a worthy point: Everything around us is intimately connected. And by studying how broad forces in nature interact, we can construct predictions that offer great benefits to society.

Now a global effort is under way to revolutionize our understanding of the Earth as an interconnected whole. The effort aims to integrate Earth observing capabilities based on satellites and *in situ* or ground-based sensors into a Global Earth Observation System of Systems (GEOSS). By uniting these systems, scientists hope to take the pulse of the planet, and in so doing, generate a range of environmental, economic, and health benefits.

For instance, should the effort yield even a 1°F improvement in weather forecasting, power utilities can plan their daily output needs more accurately, resulting in an annual $1 billion electricity savings for consumers in the United States alone, according to the U.S. Environmental Protection Agency (EPA). Likewise, improved monitoring of air pollution, or better satellite mapping of habitats that harbor malaria, cholera, or West Nile virus, could save many lives by establishing warning systems for at-risk populations that might reduce exposure.

A total of 54 countries, the European Union, and 33 international organizations have joined the GEOSS thus far, providing a welcome boost to the environmental reputation of its sponsor: the United States. The project is also the first project of its kind to get such high-level support, says Steve Goodman, chief of the Earth and Planetary Science Branch at the National Aeronautics and Space Administration (NASA) Marshall Space Flight Center.

“I’ve never seen a program move at a pace like this with such a sustained effort,” says Gary Foley, who is director of the EPA National Exposure Research Laboratory. “It seems to be the right thing at the right time with the right leadership. It was what everyone seemed to be looking for.”

## A Basis in Climate Change

The pioneering force behind the 15-agency U.S. effort is Conrad C. Lautenbacher, a retired Navy vice admiral who currently heads the National Oceanic and Atmospheric Administration (NOAA). Lautenbacher’s efforts to promote Earth observations date back to his days with the Consortium for Oceanographic Research and Education, a Washington, D.C.–based advocacy group that he directed until arriving at NOAA in 2001.

Lautenbacher’s initial goal with the Consortium for Oceanographic Research and Education was to improve studies of climate change, which he says are severely limited by data gaps and inconsistencies in ocean monitoring. Lautenbacher attributed the data shortages to what he calls a “principal investigator mentality” in ocean research. “You have research by scientists who compete for grants, publish their results, and move on,” he says. “There’s no sustainable component to it, nothing to tell you what’s going on over the long term. That’s what’s essential for climate research.”

The best way to bolster ocean monitoring, Lautenbacher reasoned, was with a sustained and globally integrated research effort based on satellites and sensing devices in the ocean that would generate continuous data streams. Such an approach, he says, would clarify the role of the oceans in climate and provide immediate benefits in coastal management.

At NOAA, Lautenbacher continued to push for an ocean observing system, but his views on the technology had begun to expand. While attending the World Summit on Sustainable Development, held in Johannesburg in September 2002, he was exposed to other uses for Earth observations in areas such as agriculture, forestry, and public health. Many scientists at Johannesburg were convinced that Earth observations are necessary to promote sustainable development across the spectrum of human activities.

When he returned to the United States, Lautenbacher and his cochairs on the National Science and Technology Council Committee on Environmental Natural Resources convened an interagency task force and charged it with organizing a global summit on Earth observations. When the Earth Observation Summit was held in Washington on 31 July 2003, it was on a scale that few of its organizers might have anticipated—five U.S. cabinet members and ministers from 33 countries and the European Union were in attendance. U.S. secretary of state Colin Powell launched the meeting, saying in his opening presentation, “We need to be able to see, hear, taste, smell, and measure the blue orb we have been given and that we call Earth.”

Participants at the summit adopted a declaration calling for a comprehensive, coordinated, and sustained Earth observation “system of systems.” Among the goals laid out in the declaration are improved coordination of Earth observing strategies and systems, the free-flowing exchange of observational data, and coordinated efforts to promote the access of developing countries to the technology. The ad hoc Group on Earth Observations (GEO) was formed and charged with creating a 10-year implementation plan to realize these goals. The cochairs of the ad hoc group now include Lautenbacher; Akio Yuki, the Japanese deputy minister of education, culture, sports, science, and technology; Achilleas Mitsos, director-general of research with the European Commission; and Robert Adam, South Africa’s director-general of science and technology.

A framework for GEOSS’s 10-year implementation plan was adopted at the second Earth Observation Summit, held in Tokyo on 25 April 2004. The completed 10-year plan will be adopted by ministers at a third summit, to be held in Brussels on 16 February 2005.

## The Nuts and Bolts

So, what exactly is an “Earth observing system”? In general, the term describes any collection of tools used to take measurements of air, water, and land. These tools can be as simple as a pH sensor or as complex as a constellation of satellites in space. Both simple and complex tools are necessary; orbiting satellites cover broad sections of the planet with limited resolution whereas ground-based tools cover more limited regions with high resolution. When combined, these technologies provide the data needed to understand how physical and biological forces control the biosphere.

A total of 73 Ear th observing satellites are in orbit now, of which 25 are owned by the United States. Of these, most are deployed by NASA, with the remainder operated by NOAA, the U.S. Geological Survey, and a few private firms. A growing number of countries—among them the European Union countries, India, Russia, China, Brazil, Japan, and Canada—also have Earth observing satellites.

Satellites gather data by remote sensing, a process that generates measurements of Earth surface features and the atmosphere according to how they reflect visible or infrared radiation. Every object—down to a single molecule—reflects radiation according to a specific wavelength, which becomes the object’s own *de facto* signature. Computer algorithms convert these signatures into measures of forest cover, soil moisture, cloud cover, ocean chlorophyll content, and many other useful parameters.

Remote sensing is an especially powerful capability when it is incorporated into a geographic information system. These systems integrate satellite and other geophysical data with socioeconomic data such as population demographics. What results are maps that allow researchers to “see” their parameter of interest in relation to their geographic position.

Although some satellite sensors can view the Earth’s surface through cloud cover, experts say that spatial and temporal resolution are still inadequate, and there is no way to image what lies underwater. Thus, it’s still necessary to use a range of additional *in situ* tools, such as weather balloons, aircraft, pollution sampling devices, and buoys that take physical measurements of the atmosphere, ocean, and land.

Fixed buoys, which are moored to the sea floor, float on the ocean surface and transmit ocean and weather data. Of the roughly 100 fixed buoys now in existence, most are found in equatorial waters. So-called Argos buoys function in a different way. These buoys sink to a depth of 2,000 meters and then drift for 10 days. Upon rising to the surface, they transmit water temperature and salinity data into space, where it is received by satellites that beam it back to scientists and weather agencies on Earth.

A total of 1,500 Argos buoys are now at work throughout the world; a global consortium responsible for the effort plans to deploy 1,500 more over the next several years.

## Establishing the GEOSS

The problem with current Earth observing systems, experts say, is that they operate in isolation without “speaking” to each other. GEOSS members often describe the systems as “research stovepipes,” with limited flexibility and focused application to particular needs. “For instance, some weather satellite instruments look only at cloud temperatures, but these same instruments, if tuned differently, could provide better detection of wildfires,” explains Helen Wood, senior advisor for systems and services in NOAA’s National Environmental Satellite, Data, and Information Service, and director of the GEO Secretariat, the forum through which GEOSS members will organize their work. One of the GEOSS’s main goals, she says, is simply to get researchers talking to each other and to end users about what they do. Just by discussing mutual needs, researchers might be prompted to design enhanced instruments that can be used for multiple purposes, she says.

Another important GEOSS goal, Wood adds, is to establish a coordinated system of data sharing that is freely accessible to users throughout the globe. Achieving this aim won’t be easy—many countries differ with respect to data standards, formats, and their own commercial views on the technology. For instance, the United States provides much of its satellite data for free, but the European Space Agency often charges for its data to recoup investment costs. Lautenbacher emphasizes that the need for consensus on business models for data sharing is critical and will likely take years to sort out.

Just the fact that GEOSS members agreed in principle to full and open data sharing with minimal time delays (as described in the 2003 declaration) was a big step forward, says Rick Ohlemacher, manager of environmental systems at Northrop Grumman Space Technology. “That in and of itself was a huge political milestone,” he says. “For several decades, no one was able to get beyond that, so now the door is open.” Northrop Grumman is one of the founding members of the Alliance for Earth Observations, an industry group established to support the GEOSS vision. The company is also the prime contractor for the future National Polar-Orbiting Operational Environmental Satellite System, which Ohlemacher says will be a key component to the satellite backbone of the GEOSS.

What the GEOSS provides, Ohlemacher says, is an enabling landscape where members can discuss how to expand global use of Earth observing technology. Ideally, the GEOSS would serve Earth observations in much the same way that the World Meteorological Organization serves weather forecasting, many experts suggest. The latter organization is an optimal model for the GEOSS for several reasons, Lautenbacher explains. It has a relatively long history (dating back to 1950), an effective governance structure, a permanent secretariat, and a mechanism for building and maintaining agreements. It also provides nations a way to manage technical issues while respecting each other’s sovereignty. “There’s no hint of language that says ‘we’re going to come into your country and tell you what to do,’” Lautenbacher says. “But you can go anywhere in the world and get a weather forecast.”

## Capacity Building

To achieve similar value, the GEOSS must not only stimulate dialogue and open access, but also must find a way to bring poorer nations into the technology loop and get the developing world actively engaged in the system. Most developing countries lack the resources needed to use Earth observations effectively. Moreover, Ohlemacher says, it can be hard for scientists in these countries to communicate the benefits of Earth observations to more traditional populations, who might not understand how information derived from space could be useful or even desirable.

The lack of developing country resources also extends to coverage; indeed, much of the developing world is a blind spot for Earth observations, says Goodman. “We have geostationary weather satellites ringing the planet,” says Goodman, “but what we need is greater temporal sampling from [higher-resolution] lower-orbiting satellites that pass over a point on Earth much less frequently —two to four times per day—to achieve the needed global coverage.” Goodman says the gaps in surface measurements are significant in the developing world—where there is less Earth-based monitoring—compared to European nations and the United States.”

The gaps are problematic because without surface observations, scientists can’t adequately validate measurements made from space. Furthermore, some parameters are not well observed from space. One example is rainfall volume, which satellites are not yet able to measure directly. In Europe and North America, thousands of simple “tipping buckets” measure minutely to hourly rainfall, which is combined with satellite remote sensing to model weather patterns and local hydrology. But tipping buckets are rare in developing countries, as are more sophisticated instruments like weather radar, which detects clouds and precipitation. “There are possibly as few as three weather radar in the entire African continent,” Goodman says. “Here, every state has one, and TV stations tend to also have their own.”

Global blind spots have numerous consequences. Obviously, without sufficient coverage, developing nations don’t have access to global observation data that might be useful to them [for more on this topic, see “Building a Tsunami Warning System,” p. A90 this issue].

But the consequences also extend to developed countries and the Earth as a whole. Goodman points out that long-term weather forecasts in North America are less accurate than they could be thanks to a lack of data from the oceans as well as from Asia, Africa, and Latin America. Moreover, studies of long-term trends such as climate change depend on knowledge of events taking place throughout the biosphere. If scientists can’t quantify the effects of, say, carbon fluxes in the Amazon basin, they will be unable to generate the more comprehensive climate models they desire.

## Earth Observation in Action

And yet, scientists interviewed for this article unanimously agree that Earth observations are useful now, and are becoming more so. Michael Emch, a spatial epidemiologist at Columbia University, has spent years using remote sensing to study tropical disease epidemiology. Much of his work is devoted to studies of cholera in the coastal nations of Bangladesh, Vietnam, and Mozambique. Using remote sensing data from NASA’s Terra and Aqua satellites—which are both components of the agency’s Earth Observing System program—Emch has correlated cholera incidence with sea surface height, sea surface temperature, and ocean chlorophyll content. These parameters are all potentially linked to the generation of plankton blooms that harbor *Vibrio cholerae*, the bacterium that causes cholera. “If we can sort out the role of these variables, then we might be able to predict epidemics months before they occur,” he says. “That’s the ultimate goal, but it’s a long way off.”

Emch concedes there is an ever-growing need for new instrumentation. For instance, satellites are unable to detect the salinity levels that are key to the survival of *V. cholerae*. Greater image resolution is also needed to quantify and monitor environmental changes, he says.

The multichannel remote sensing device carried by the Terra and Aqua satellites, which is known as the Moderate-Resolution Imaging Spectroradiometer, or MODIS, measures ocean chlorophyll levels in spatial increments of 1,000 meters and land boundary changes at increments of 250 meters [for more information on this instrument, see “MODIS Operandi for Mapping Haze,” *EHP* 111:A458 (2003)].

This is a significant improvement over previous sensing technologies, but one that still falls short of some research needs. In most cases, remote sensing won’t provide conclusive evidence that an outbreak is going to occur, Emch says. Scientists also need ground-based observations—for instance, of the micro-scale environments inhabited by disease vectors, or of actual human populations at risk.

Another active proponent of Earth observing technology is Foley, who says Earth observations are increasingly relevant to the EPA’s mission. For more than 10 years, the EPA has used remote sensing data generated by NASA’s Landsat satellite program (now in its 33rd year) to monitor changes in urban landscapes, among other uses. Remote sensing will ideally advance new public warning systems for air and water pollution hazards, Foley says. But in the meantime, his biggest challenge is convincing EPA decision makers of the technology’s potential. “They often don’t know how to use the data,” he explains. “It’s not traditional, so we need to do some education and capacity building right here. But we intend to go all the way with this—resources, time, and science permitting.

John Delaney is as close to the technology’s forefront as anyone on Earth. A professor of oceanography at the University of Washington in Seattle, Delaney directs the Northeast Pacific Time-Series Undersea Networked Experiments (NEP-TUNE) Program, one of the most comprehensive Earth observing efforts ever launched. NEPTUNE scientists seek to wire an entire tectonic plate off the Pacific Northwest with thousands of sensors both above and below the sea floor, all of them delivering real-time data to scientists on shore. The plan also calls for a fleet of underwater robots that will travel toward volcano eruptions, earthquakes, storms, and any number of other events to collect data. The $250 million price tag for the system is gradually being met through a variety of grant programs.

“[This program] gives us the ability to be in the environment continuously,” Delaney says. “To really understand the ocean we have to be in the environment all the time and be flexible enough to adapt, observe, quantify, and sample.” Lautenbacher describes NEPTUNE as being on the cutting edge of research. “It’s where we will develop new sensors and ways to measure things we can now only dream of,” he says.

## Moving Forward

In the years to come, GEOSS stakeholders will be focused on a number of priorities. According to Lautenbacher, these include the creation of a governance structure for the organization, the resolution of technical issues related to data sharing, and an inventory of Earth observing capacity needs.

Meanwhile, many of the countries working toward the GEOSS are also pursuing systems integration efforts at home. The United States, for instance, has convened an Interagency Working Group on Earth Observations, which is cochaired by representatives from NASA, NOAA, and the White House Office of Science and Technology Policy.

On 8 September 2004, the group released a draft version of its *Strategic Plan for the U.S. Integrated Earth Observation System*. The report lays out a framework for U.S. participation in the GEOSS and describes opportunities in nine “societal benefits areas” where the technology can advance environmental goals.

Mary Gant, a program analyst at the NIEHS and a member of the interagency working group, says Earth observations will be broadly useful for environmental health, and for exposure assessment in particular. “One of the most difficult problems with which the health communities must cope is the accuracy of exposure assessment,” she says. “The data and data products from an enhanced, integrated Earth observation system should greatly increase our ability to do exposure assessment.”

Other countries are also proceeding with their own domestic and regional programs, albeit inconsistently, Lautenbacher says. In terms of countries that could propel the GEOSS, those represented by the European Union are perhaps best prepared, he suggests. The European Union has a Global Monitoring for Environment and Security initiative, which has created partnerships among the European Space Agency, the European Environment Agency, and other institutions. The initiative lays out a process for ensuring that Earth observing data needs are met in both the near and long terms.

Initiative members are now establishing a uniform architecture for integrating space, terrestrial, seaborne, and airborne monitoring platforms. Thus, the initiative may provide a model for technical integration throughout the GEOSS, Lautenbacher says.

Earth observations could do much to improve our views and our understanding of the world around us. Many challenges remain for GEOSS, not just in terms of technical barriers but also cultural and institutional ones. But this growing network of sensors and satellites may represent the best opportunity to discover and quantify the fundamental drivers of the biosphere. Just as satellites and space exploration programs turn our attention outward, similar tools focus our attention inward, toward the unifying forces that hold life in a balance.

## Figures and Tables

**Figure:**
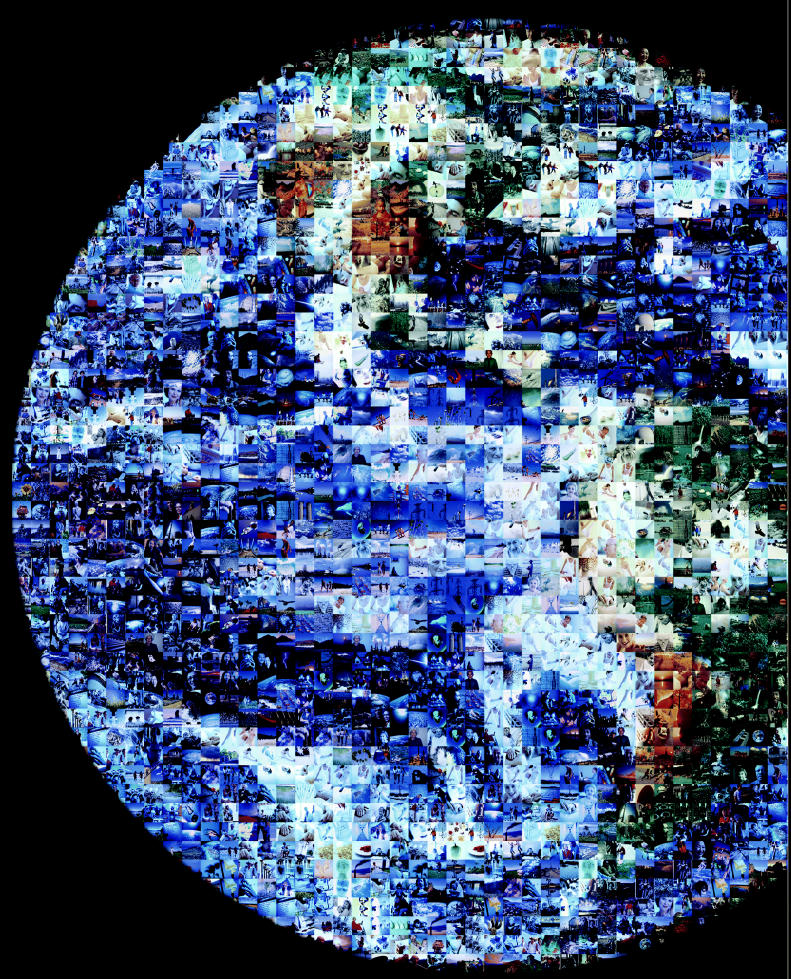


**Figure:**
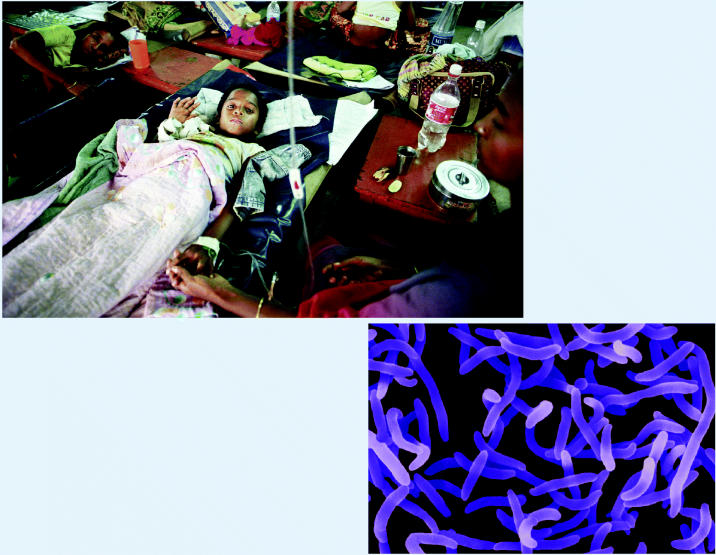
**A system for change.** Proponents of a new Global Earth Observation System of Systems (GEOSS) envision using data from satellites and a variety of ground-based sensors to aid public health goals around the world. One potential use is to compile satellite mapping of habitats that harbor disease microbes such as *Vibrio cholerae* (right). This may help provide early warning to at-risk populations, such as the residents of Dhaka, Bangladesh, where one hospital alone (above) received more than 300 new cholera patients every day during monsoon-related flooding in July and August 2004.

**Figure:**
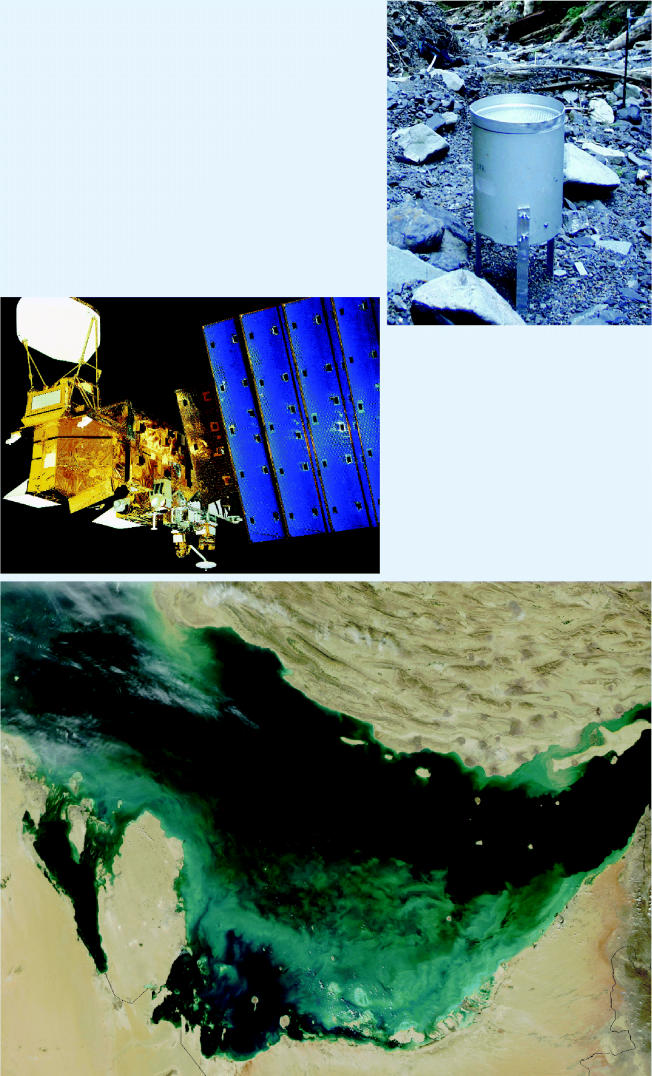
**It takes all kinds . . . of tools.** To be fully functional, the GEOSS will rely on a variety of tools. Simple gauges such as tipping buckets (above), which measure rainfall, provide measurements that can be correlated with data from more complex technologies, such as the satellite Aqua (left). The satellite image below, taken with MODIS instrumentation aboard Aqua, shows clouds of sediment (pictured in turquoise) in the southern Persian Gulf mixed with microscopic marine organisms (where the clouds appear more greenish). Such information may offer clues about potential future disease outbreaks.

**Figure:**
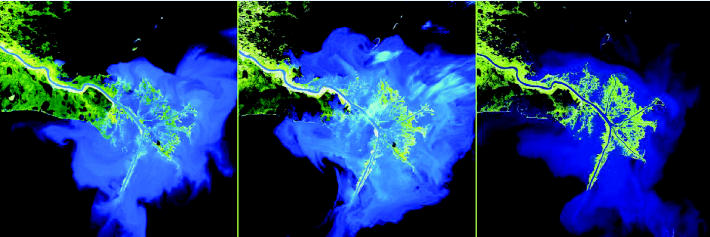
**The delta blues tell a story.** Data gathered over the long term may help researchers better predict how Earth systems will change in the future. The Landsat images above show how sedimentation of the Mississippi Delta has changed over the past 30 years. The first image, taken in 1973, shows a rich swell of sediment (visualized in blue). But the building of dams and artificial channeling along the Mississippi–Missouri River system decreased the amount of sediment that the currents carry; the second image, taken in 1989, shows how the delta began to lose marshland along its southeast edge. By 2003, when the third shot was taken, the addition of channels in the natural river levee had resulted in new marsh formation.

**Figure:**
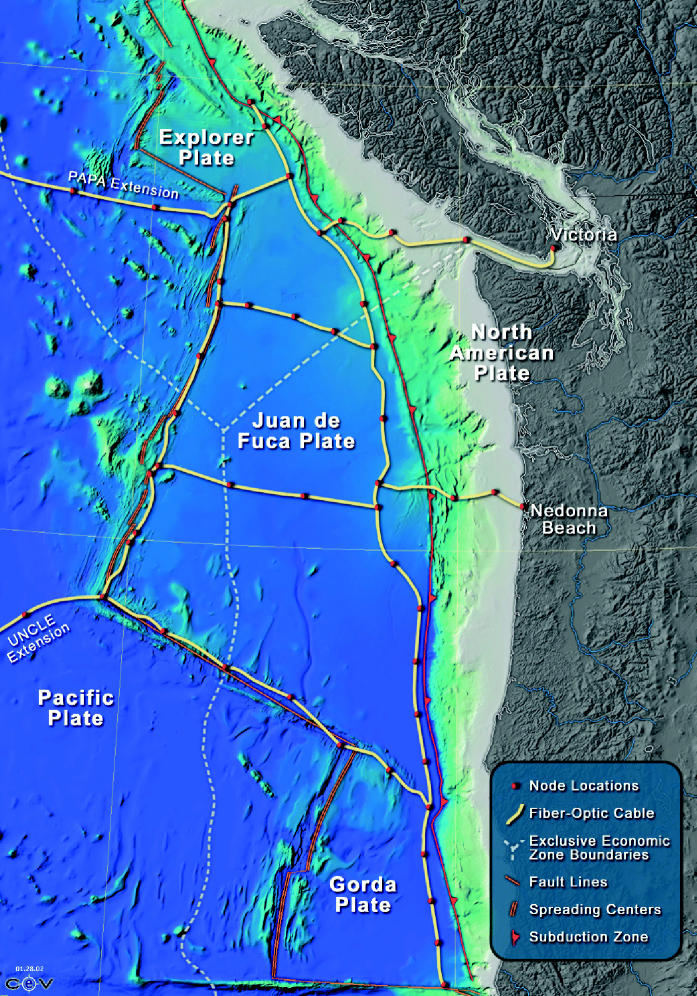
**Poseidon adventure?** The Northeast Pacific Time-Series Undersea Networked Experiments (NEPTUNE) Program is an ambitious plan to wire an entire tectonic plate off the Pacific Northwest with sensors delivering real-time data from above and below the sea floor.

